# Multi-Strategy Enhanced Parrot Optimizer: Global Optimization and Feature Selection

**DOI:** 10.3390/biomimetics9110662

**Published:** 2024-10-31

**Authors:** Tian Chen, Yuanyuan Yi

**Affiliations:** College of Geophysics and Petroleum Resources, Yangtze University, Wuhan 430100, China; tianchen_ytu@163.com

**Keywords:** parrot optimizer, global optimization, feature selection, crisscross mechanism, metaheuristic algorithms, bionic algorithm

## Abstract

Optimization algorithms are pivotal in addressing complex problems across diverse domains, including global optimization and feature selection (FS). In this paper, we introduce the Enhanced Crisscross Parrot Optimizer (ECPO), an improved version of the Parrot Optimizer (PO), designed to address these challenges effectively. The ECPO incorporates a sophisticated strategy selection mechanism that allows individuals to retain successful behaviors from prior iterations and shift to alternative strategies in case of update failures. Additionally, the integration of a crisscross (CC) mechanism promotes more effective information exchange among individuals, enhancing the algorithm’s exploration capabilities. The proposed algorithm’s performance is evaluated through extensive experiments on the CEC2017 benchmark functions, where it is compared with ten other conventional optimization algorithms. Results demonstrate that the ECPO consistently outperforms these algorithms across various fitness landscapes. Furthermore, a binary version of the ECPO is developed and applied to FS problems on ten real-world datasets, demonstrating its ability to achieve competitive error rates with reduced feature subsets. These findings suggest that the ECPO holds promise as an effective approach for both global optimization and feature selection.

## 1. Introduction

In the era of data-driven decision-making, where vast amounts of data are generated daily, the need to extract meaningful information efficiently becomes paramount. One crucial aspect of this extraction process is feature selection (FS), which involves identifying and choosing the most relevant and informative features from a potentially overwhelming set of data [1]. FS is vital for several reasons. Firstly, in the realm of machine learning, models trained on irrelevant or redundant features can suffer from decreased accuracy and efficiency [2]. These extraneous features can introduce noise, complicating the learning process and potentially leading to overfitting. By carefully selecting the most pertinent features, we can streamline the model, enhancing its predictive power and generalizability. Secondly, feature selection bolsters the interpretability of machine learning models [3]. In many applications, especially those in healthcare, finance, or policy-making, understanding which features contribute most significantly to the model’s output is essential [4]. By reducing the feature set, we can more clearly discern the impact of each variable, thereby facilitating transparent and accountable decision-making [5].

FS techniques can be broadly categorized into three main approaches: wrapper, filter, and embedded methods [6]. Wrapper methods involve evaluating various feature subsets based on their impact on a specific learning algorithm’s performance [7]. Common examples include recursive feature elimination (RFE) [8] and evolutionary algorithms (EAs) [9], which iteratively assess the contribution of features to the model’s accuracy. Filter methods [10], on the other hand, rely on statistical properties to assess the relevance of individual features, independent of any learning algorithm. Techniques such as the chi-square test [11], information gain [12], and correlation coefficient [13] are widely used in this category. Embedded methods integrate the feature selection process into the model training phase, allowing for simultaneous learning and feature selection. Methods like L1 regularization (e.g., Lasso regression) and tree-based models that provide feature importance rankings (e.g., random forest) fall under this category [14].

While these traditional methods have proven effective in various scenarios, they often face challenges when dealing with complex, high-dimensional datasets. Metaheuristics offer a promising alternative for feature selection. These algorithms excel in exploring the vast search space efficiently, identifying globally optimal feature subsets [15]. Their inherent ability to handle diversity, avoid local minima, and adapt to changing landscapes makes them particularly suitable for complex optimization tasks, including feature selection in high-dimensional data.

Metaheuristic algorithms are generally categorized into two types: evolutionary algorithms and swarm intelligence optimization algorithms. Evolutionary algorithms, exemplified by the genetic algorithm (GA) [16], mimic the natural evolutionary process through selection, crossover, and mutation operations to evolve better solutions over generations. On the other hand, swarm intelligence optimization algorithms, such as particle swarm optimization (PSO) [17], draw inspiration from the collective behavior of social insects or animals. These algorithms utilize the concept of swarm intelligence to search for the global optimum through collaboration and information sharing among individuals.

Over the past few years, many scholars have dedicated their efforts to solving the FS problem using metaheuristic algorithms [18]. Shan et al. [19] proposed a multi-strategy enhanced crow search algorithm. The proposed method effectively improved the optimization performance of the algorithm through a crossover mechanism and a combined mutation mechanism. FS experiments were conducted on 10 datasets using the proposed method, and the results indicated that the proposed approach could obtain feature subsets with higher accuracy. Tubishat et al. [20] proposed an enhanced butterfly optimization algorithm for feature selection, termed DBOA, which incorporates a local search algorithm based on mutation to overcome local optima and improve solution diversity. Tested on 20 UCI datasets, the DBOA outperformed several benchmark algorithms in classification accuracy and feature subset selection efficiency. Kwakye et al. (2024) [21] proposed a particle swarm-guided bald eagle search (PS-BES) algorithm for global optimization and feature selection. The algorithm introduced the Attack–Retreat–Surrender technique to enhance balance between diversification and intensification. The study demonstrated the superior performance of the PS-BES through comprehensive evaluations using 26 benchmark functions and 27 classification datasets from the UCI repository, comparing it favorably against ten state-of-the-art algorithms. Abdelrazek et al. [22] proposed a modified version of the dwarf mongoose optimization algorithm named CDMO for feature selection. This new approach integrated ten chaotic maps to enhance the DMO’s convergence speed and effectiveness. Numerous studies have been conducted on metaheuristic algorithms for FS problems, and similar research is thoroughly reviewed in the literature [23].

Despite the development of such metaheuristic algorithms for feature selection research, the “no free lunch” theorem indicates that no single algorithm can perform well on all datasets, urging us to develop more algorithms to solve different problems.

The parrot optimizer (PO) is a novel metaheuristic algorithm proposed by Lian et al. in 2024 [24], inspired by the foraging, dwelling, communication, and fear of strangers exhibited by domesticated Green-cheeked parakeet (Pyrrhura molinae). Unlike the traditional exploration–exploitation two-stage structure, the PO effectively escapes local optima by randomly adopting four different behaviors for each individual. However, the PO still suffers from some problems that lead to its slow convergence due to its randomly chosen search strategy; it often makes the search stagnant by generating inferior solutions.

In this paper, we present an enhanced version of the parrot optimizer (PO), named ECPO. By improving its strategy selection mechanism, the ECPO enables each individual to repeat the behavior that successfully updates its position in the previous iteration, and to randomly switch to another behavior upon failure, thereby enhancing the algorithm’s search capabilities. The introduction of a crisscross (CC) mechanism strengthens the exchange of information among individuals, further augmenting the algorithm’s exploration abilities. Additionally, we propose a binary version of the ECPO, which is applied to feature selection problems on real-world datasets. The main contributions of this paper are as follows:

- An enhanced PO algorithm is proposed by introducing the CC mechanism and enhanced strategy selection mechanism.
- The performance of the ECPO algorithm is verified in detail, through comparison experiments with 10 other conventional optimization algorithms on the CEC2017 benchmark functions.
- A binary version of the ECPO for solving FS problems and validated on ten real datasets that the ECPO can effectively solve the FS problems.

The organization of this paper is as follows: Section 1 provides an introduction to the research background and motivation, includes a brief literature review, and concludes with a summary of the paper’s main contributions. Section 2 outlines the original PO algorithm. In Section 3, the CC mechanism and enhanced strategy selection mechanism are described in detail, along with the proposed ECPO algorithm. Section 4 covers the methodology, results, and analysis of the global optimization experiments. Section 5 details the experiments related to feature selection on ten real-world datasets. Finally, Section 6 wraps up the paper with a summary.

## 2. The Original PO

The PO is a new metaheuristic optimization algorithm inspired by the behavior of Pyrrhura molinae parrots, proposed by Lian et al. in 2024 [24]. The PO aims to solve complex optimization problems by mimicking the foraging, perching, communicating, and fear of strangers behaviors observed in these parrots. By modeling the four behaviors of parrots, the optimization process of the algorithm is divided into the following four stages:

1. Foraging behavior: In the foraging behavior of the PO, the approximate location of food is determined primarily through observation of the food’s position or by considering the owner’s location, and subsequent flight towards that position. The positional movement is governed by Equation (1).

(1) $$X_{i}^{t+1}=(X_{i}^{t}-X_{best})\cdot Levy(dim)+rand(0,1)\cdot (1-\frac{t}{Max_{iter}}{)}^{\frac{2t}{Max_{iter}}}\cdot X_{mean}^{t}$$

where $X_{best}$ denotes the optimal solution obtained by the current iteration, $X_{i}^{t}$ denotes position of the ith particles at the tth iteration, $X_{i}^{t+1}$ denotes the position that will be updated in the next iteration, $X_{mean}^{t}$ donotes the average position of the population at the tth iteration. Levy(dim) represents a Lévy distribution of the problem’s dimensionality, and t and $Max_{iter}$ represent the current iteration and maximum number of iterations, respectively.

2. Staying behavior: In the staying behavior of the PO, the behavior of the particle is modeled by simulating a parrot randomly landing on any position of its owner and remaining stationary. Progress is governed by Equation (2).

(2) $$X_{i}^{t+1}=X_{i}^{t}+X_{best}\cdot Levy(dim)+rand(0,1)\cdot ones(1,dim)$$

where ones(1,dim) is a 1xdim all-ones matrix, and this process signifies the parrot flying towards its owner and randomly stopping.

3. Communicating behavior: In the communicating behavior of the PO, the movement process of particles simulates information exchange within a parrot flock, manifesting specifically in two behaviors: flying towards the flock and moving away from it. In PO, it is assumed that these two behaviors occur with equal probability, and the average position of the current population is used to represent the center of the flock. This process is modeled in Equation (3).

(3) $$X_{i}^{t+1}=\{\begin{array}{cc} 0.2\cdot rand(0,1)\cdot (1-\frac{t}{Max_{iter}})\cdot (X_{i}^{t}-X_{mean}^{t}) & P\leq 0.5\\ 0.2\cdot rand(0,1)\cdot exp(-\frac{t}{rand(0,1)\cdot Max_{iter}}) & P>0.5 \end{array}$$

In the formula, the part where p≤0.5 is used to achieve individual approach towards the center of the population, while the part where p>0.5 is used to generate random positions, enabling the individual to move away from the population.

4. Fear of strangers behavior: In fear of strangers behavior of the PO, particles simulate the behavior of maintaining distance from unfamiliar individuals and seeking a safe environment together with the owner, as shown in Equation (4).

(4) $$X_{i}^{t+1}=X_{i}^{t}+rand(0,1)\cdot \cos(0.5\pi \cdot \frac{t}{Max_{iter}})\cdot (X_{best}-X_{i}^{t})\;\;-\cos(rand(0,1)\cdot \pi )\cdot (\frac{t}{Max_{iter}}{)}^{\frac{2}{Max_{iter}}}\cdot (X_{i}^{t}-X_{best})$$

In the formula, the upper part represents the process of adjusting the direction to fly towards the owner, while the lower part represents the process of moving away from strangers.

During the initialization phase of the algorithm, a set of predefined solutions are generated as the initial population through Equation (5).

(5) $$X_{i}^{0}=lb+rand(0,1)\cdot (ub-lb)$$

where ub and lb represent the upper and lower bounds, respectively. $X_{i}^{0}$ is the initial population generated.

**Figure 1:**
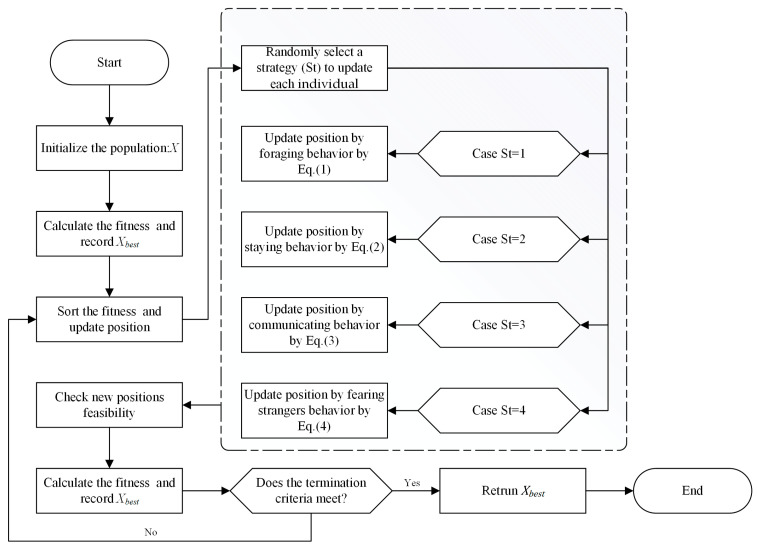
Flowchart of the PO.

Afterwards, iterative updates begin. In the updating process, the algorithm will randomly select several stages from the four stages to update individuals in the population. Each solution will be dynamically adjusted based on the best solution identified by the PO algorithm so far. The entire updating process will continue to iterate until the maximum number of iterations is reached. A flowchart for the PO is shown in Figure 1.

## 3. Proposed ECPO

### 3.1. Crisscross Strategy

The design of the crossover strategy draws inspiration from Meng’s crossover optimizer (CSO) [25]. The approach consists of two main parts, horizontal crossover search (HCS) and vertical crossover search (VCS), which enable the exchange of horizontal and vertical information between particles, respectively. This crossover strategy focuses on generating new particles by exchanging information among randomly selected particles or dimensions. The most fit particles are preserved and added back into the population, thereby enhancing the global search capability of the crossover mechanism. Shan et al. [19] improved the CSA by adding a crossover strategy along with a combined mutation approach, which assists the population in avoiding local optima. Similarly, Hu et al. [26] incorporated the crossover strategy into the SCA algorithm, revealing that it boosted global convergence, enhanced population diversity, and supported the escape from local optima.

In this study, we introduce the CC strategy in the original PO and improve its strategy management mechanism to propose the ECPO. The CC strategy and the improved strategy management mechanism are described in detail below.

#### 3.1.1. Horizontal Crossover Search

The HCS will randomly select the particles in the population and randomly pair two by two to perform the crossover operation. The HCS can make more use of the population information and improve the exploration ability of the algorithm. The HCS operation is defined by Equations (6) and (7).

(6) $${HCS}_{i}^{j}=r_{1}\times x_{i}^{j}+\left(1-r_{1}\right)\times x_{k}^{j}+c_{1}\times \left(x_{i}^{j}-x_{k}^{j}\right)$$

(7) $${HCS}_{k}^{j}=r_{2}\times x_{k}^{j}+\left(1-r_{2}\right)\times x_{i}^{j}+c_{2}\times \left(x_{k}^{j}-x_{i}^{j}\right)$$

where $r_{1}$ and $r_{2}$ are random numbers in interval [0, 1], $c_{1}$ and $c_{2}$ are random numbers in interval [−1, 1], $x_{i}^{j}$ is the value of the jth dimension of the ith particle, $x_{k}^{j}$ is the value of the jth dimension of the kth particle. ${HCS}_{i}^{j}$ and ${HCS}_{k}^{j}$ are the new offspring of the two particles generated by the HCS. HCS ends up performing greedy selection to retain individuals with better fitness values between offspring and parents. Algorithm 1 presents the pseudo-code for the HCS operation.
**Algorithm 1** HCSRandIndex = randperm (n) **For**
i=1:n/2
  i = RandIndex (2i−1) k = RandIndex (2i)   **For** j=1:dim    Generate four random number $r_{1}$, $r_{2}\in$(0,1), $c_{1}$, $c_{2}\in$(−1,1)    Generate ${HCS}_{i}^{j}\;$and ${HCS}_{k}^{j}\;$by Equations (6) and (7)  **End** For **End**
For **For**
i=1:n  **IF** F(${HCS}_{i}$) < F($X_{i}$)                $X\left(i\right)=HCS(i)$  **End** IF **End** For **End**

#### 3.1.2. Vertical Crossover Search

The VCS will perform a crossover operation on two random dimensions of each particle. Similarly, the VCS ends up with perform greedy selection to retain individuals with better fitness values between offspring and parents. The VCS operation is defined by Equation (8).

(8) $${VCS}_{i}^{j}=r_{3}\times x_{i}^{j1}+\left(1-r_{3}\right)\times x_{i}^{j2}$$

where $r_{3}$ is a random number in interval [0, 1], $x_{i}^{j1}$ and $x_{i}^{j2}$ indicate the values of the two dimensions chosen at random by the ith individual, respectively, ${VCS}_{i}^{j}$ represents the value of the jth dimension generated from two random dimensions of the ith particle. Algorithm 2 presents the pseudo-code for the VCS.
**Algorithm 2** VCSRandIndex = randperm (dim) Generate a random number p∈(0,1) **For**
j=1:dim/2   **IF** p < 0.6
   j1 = RandIndex (2j−1)   j2 = RandIndex (2j)   **For** i=1:n       Generate a random number $r_{3}\in$(0,1)       Generate ${VCS}_{i}^{j}$ by Equation (6)   **End** For   **End** IF **End**
For **For**
i=1:n   **IF** F(VCS(i)) < F(X(i))        $X\left(i\right)=VCS(i)$
   **End** IF **End**
For **End**

### 3.2. Enhanced Strategy Management

The original PO is randomly updated in one of four ways in each iteration and, in this paper, a greedy selection-like approach is used for the policy management of the algorithm. Specifically, the algorithm will record whether the strategy chosen each time succeeds in improving the particles; if it succeeds, it will continue to use the strategy; if it fails, it will randomly choose one of the remaining three strategies.

The greedy selection approach to policy management has faster convergence and optimization efficiency compared to a completely random selection policy. Improved strategies tend to reuse strategies that have successfully improved examples in past iterations, meaning that the algorithm is more likely to move in a better direction in the search space. By recording and prioritizing successful strategies, the algorithm is able to make more efficient use of limited computational resources.

### 3.3. The Proposed ECPO

**Figure 2:**
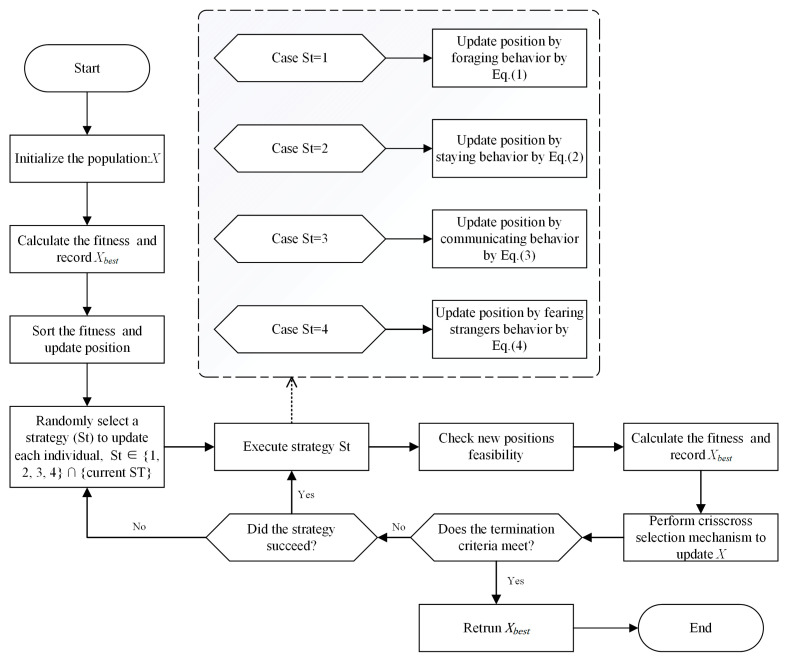
Flowchart of the ECPO.

In this subsection we describe the workflow of the ECPO. Firstly, the ECPO will initialize the parameters required for the PO and generate the initial population; after which the algorithm first randomly selects the policy as the initial policy for particle updating. After that, an improved greedy strategy management is utilized to decide the strategy to be executed by each particle. At the end of each iteration of the population update, the algorithm will execute the CC strategy to generate the new population generated after the HCS and VCS operations. This process will continue iterating until the algorithm’s termination criteria are met, at which point the optimal solution is returned. The flowchart illustrating the algorithm is presented in Figure 2.

Algorithm 3 provides the pseudo-code for the ECPO.
**Algorithm 3** Pseudo-code of the ECPOSet parameters: The maximum iteration number T, the problem dimension dim, and the population size N
Initialize population X
t = 1 St=randi([1,4]) **For** i=1:N
  Evaluate the fitness value of $x_{i}$
  Find the global min $x_{best}$
**End** For **While (**t≤T)
  **IF** St=1                       /* Behavior 1 */     Update X by Equation (1)  **ELSE IF** St=2                    /* Behavior 2 */     Update X by Equation (2)  **ELSE IF** St=3                    /* Behavior 3 */     Update X by Equation (3)  **ELSE IF** St=4                    /* Behavior 4 */     Update X by Equation (4)  **END**  $x_{old\_best}=x_{best}$           /* Enhanced Strategy Management */  Update $x_{best}$
  **IF** $x_{old\_best}=x_{best}$     St=randi([1,4])  **END IF**  **For** i=1:N                         /*CC*/     Perform Horizontal crossover search to update $x_{i}$
     Perform Vertical crossover search to update $x_{i}$
     Update $x_{best}$  **End**
For
  t=t+1; **End While**
**Return** $x_{best}$
**End**


The computational complexity of the ECPO depends on four basic aspects: population initialization, computation of fitness function, position update of particles and the CC strategy. So, the computational complexity O (ECPO) ≈ O (T × N) + O (T × N) + O (T × N × D) + O (T × N × D) ≈ O (T × N × D).

## 4. Results and Analysis of Global Optimization Experiments

This section uses the 29 benchmark functions of CEC2017 to conduct a complete evaluation of the proposed ECPO. All experiments are conducted fairly on industry-standard benchmarks. The experiments are performed on an Intel i5-13600KF with 32 GB of RAM and a Windows 11 operating system, using MATLAB 2024a for coding. In the comparative experiments, the population size of all algorithms is set to 30, the problem dimension is set to 30, the maximum number of evaluations is set to 300,000, and after running 30 times, the average and standard deviation are recorded for each function as the experimental results.

### 4.1. Benchmark Function

This subsection introduces the 29 benchmark functions used for testing, which originate from the 2017 IEEE Congress on Evolutionary Computation (CEC2017) [27]. These functions primarily consist of four types: unimodal, multimodal, hybrid, and composition functions. They are used to evaluate the performance of functions across different types of functions in detail. Table 1 provides an introduction to the function set of CEC2017.

### 4.2. Comparison of Performance with Other Algorithms

In this subsection, we compare the proposed ECPO with eight other classical algorithms on the CEC2017 benchmark functions. These algorithms include PO, SMA, WOA, MFO, BA, SCA, PSO, and DE. The hyperparameters of these algorithms are given in Table 2.

Table 3 summarizes the average fitness value (Avg) and standard deviation (Std) for the ECPO and other algorithms across each benchmark function in CEC2017. The ‘Rank’ section shows the Friedman test ranking for each algorithm, while ‘AVG’ indicates the average ranking the algorithm attained over all functions in CEC2017. The ‘+/−/=’ column illustrates whether the ECPO is better than, equal to, or worse than the other algorithms.

Table 3 indicates that the ECPO achieves an average ranking of 2.0345 on the benchmark function, placing it first among all algorithms, highlighting its considerable advantage over competitors. The ECPO reached the global optimum in all 30 trials for F5 and F8, and came close to this optimum in F2, F21, F20, F22, and F24, demonstrating the algorithm’s consistent optimization reliability. Among the compared algorithms, the DE performs closest to the ECPO but still falls short in 14 functions.

Table 4 reinforces the findings presented in Table 3. In the context of the Wilcoxon signed-rank test, a *p*-value lower than 0.05 indicates that the null hypothesis can be dismissed, showing a significant difference between the tested algorithm and the comparative algorithms. The data in Table 4 reveals that the majority of functions have *p*-values below 0.05, providing compelling evidence that the ECPO notably outperforms the other algorithms across the benchmarks.

**Figure 3:**
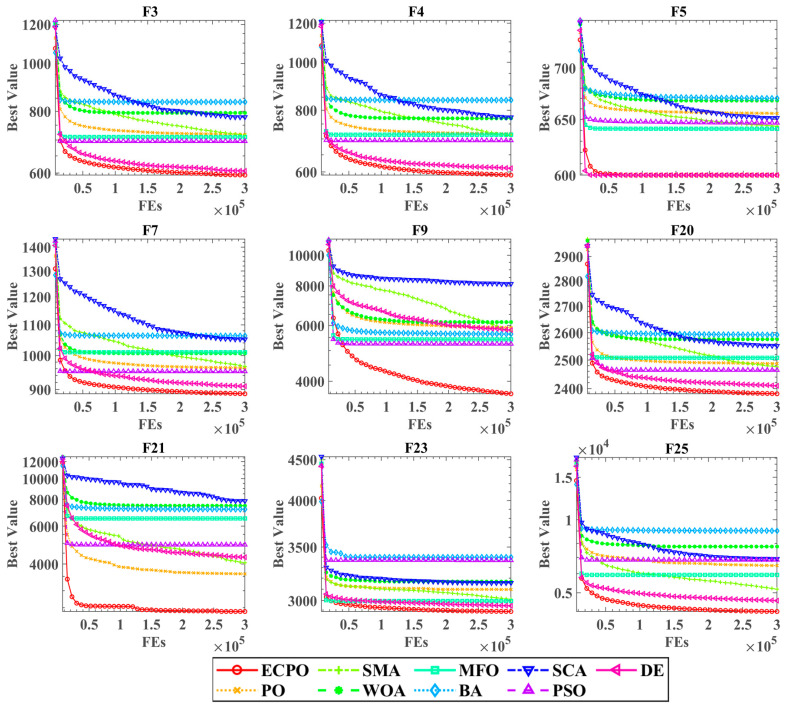
Convergence curves of the ECPO on benchmarks with other algorithms.

Figure 3 presents the convergence curves for all algorithms evaluated on specific functions. The horizontal axis denotes the number of evaluations carried out by the algorithms, while the vertical axis indicates the best fitness value achieved at any point. The legend found at the bottom of Figure 3 clarifies which algorithms are represented. Importantly, the red lines consistently fall below the other colored lines across all types of functions, which suggests that the ECPO effectively navigates away from local optima and identifies superior solutions compared to the other algorithms. In conclusion, the CC markedly enhances the search capabilities of the PO, demonstrating a significant edge over the competing algorithms in the benchmarks.

## 5. Application to Feature Selection

In this section, we apply the ECPO to feature selection and compare it with six meta-heuristics on 22 real datasets. FS is a classical combinatorial optimization problem, where whether or not to select a feature is determined by 0 and 1. Thus, each individual of the initial population of the ECPO is generated by randomly populating 0 and 1. We set the upper and lower bounds of the problem between 0 and 1 and use Equation (9) to determine whether each feature is selected or not, thus allowing the ECPO, which is only valid in the continuous domain, to act on the search space of the feature selection binary.

(9) $$X_{i,j}=\left\{\begin{array}{c} 0\;\;\;\;\;X_{i,j}<0.5\\ 1\;\;\;\;\;X_{i,j}\geq 0.5 \end{array}\right.$$

where $X_{i,j}$ denotes the jth feature of the ith subset of features in the population, Equation (9) implies that all features greater than 0.5 will be selected, while those less than 0.5 will not be selected.

The KNN is used as the evaluated classifier, and Equation (10) is used as the fitness function for feature selection

(10) $$Fitness=\mu \times E+(1-\mu )\times \frac{l}{L}$$

where E is the error rate, l is the length of the selected feature subset, and μ is a constant value between 0 and 1 used to control the weights of the feature subset length and error rate. In this experiment we are more concerned about the accuracy of the feature subset, so we set μ to 0.05.

### 5.1. Datasets Used in Experiments

In order to further evaluate the performance of the proposed method, feature selection experiments were conducted on 10 datasets selected from the UCI database, with the number of samples in the selected datasets ranging from 72 to 1473, and the feature dimensions ranging from 9 to 7130. Table 5 shows a detailed description of the datasets used, which includes the number of samples, number of classifications, and feature dimensions.

### 5.2. Analysis and Discussion of Experimental Results

This subsection presents the experimental results of the ECPO and the comparison algorithms on various datasets. The comparison algorithms include the BGWO [28], BGSA [29], BPSO [30], BBA [31], and BSSA [32]. The experiments were conducted on ten real-world datasets, with a population size of 30 and a maximum iteration count of 1000 set for each algorithm. Ten-fold cross-validation was used to avoid the contingency of the experiments. Detailed experimental results are provided in Table 6 and Table 7.

The error rate of the experiment and the average number of selected features are shown in Table 6 and Table 7. The first row for each dataset represents the mean, and the second row represents the variance. The minimum value of the mean obtained for each dataset is in bold. From the table, it can be seen that the proposed ECPO has achieved the best performance on all datasets. Notably, it excels on datasets with fewer samples, such as heartandlung and hepatitisfulldata, where the error rates are all below one percent, outperforming other algorithms by an order of magnitude. On the cmc dataset, all algorithms perform poorly with error rates above 45%, which is attributed to the performance bottleneck of the classifier. However, the ECPO has a lower error rate compared to other binary metaheuristic algorithms. On the Leukemia and Leukemia1 datasets, the ECPO, BGWO, BPSO, and BSSA all achieved an error rate of 0, while BGSA and BBA performed the worst. Specifically, the error rates of the BGSA were 10.2% and 6.31%, and the error rates of BBA were 1.67% and 5.19%.

From the perspective of the length of the selected feature subsets, the ECPO does not have an advantage over other algorithms. The BGSA tends to discover shorter feature subsets, especially on high-dimensional datasets such as Leukemia and Leukemia1, which are an order of magnitude lower than other algorithms. However, due to this, its classification error rate is relatively higher compared to other algorithms.

In summary, the ECPO has achieved the best performance among the BGWO, BGSA, BPSO, BBA, and BSSA. This is attributed to the effective enhancement of population diversity by the CC and the adaptive improvement of the algorithm population update efficiency by the greedy strategy selection mechanism.

## 6. Conclusions

In this study, we improve the strategy selection mechanism of the original PO algorithm and combine it with the CC strategy to propose an enhanced PO, where the CC strategy improves the diversity of generated offspring by enhancing the exchange of information between populations, thus accelerating the algorithm’s ability to find the best solution. The enhanced strategy selection approach maximizes the use of each update of the algorithm by adopting a greedy approach and repeatedly retaining the mechanism of successful updates. The proposed algorithm is compared with eight optimization algorithms in experiments on the CEC2017 benchmark function, and the experimental results show that the ECPO is able to achieve better performance than the other algorithms on functions with different types of fitness landscapes.

In addition, the ECPO is also used to solve the feature selection problem under 10 datasets using KNN as an evaluator, and the experimental results show that ECPO can achieve lower error rates with shorter feature subsets and is a competitive method for feature selection.

In our future research, we plan to develop and explore more advanced optimization methods. In addition we will explore the combination of deep learning and reinforcement learning techniques with optimization algorithms to solve real-world optimization problems more efficiently and intelligently.

## Figures and Tables

**Table 1:** CEC2017 benchmark functions.

Function	Function Name	Class	Optimum
F1	Shifted and Rotated Bent Cigar Function	Unimodal	100
F2	Shifted and Rotated Zakharov Function	Unimodal	300
F3	Shifted and Rotated Rosenbrock’s Function	Multimodal	400
F4	Shifted and Rotated Rastrigin’s Function	Multimodal	500
F5	Shifted and Rotated Expanded Schaffer’s F6 Function	Multimodal	600
F6	Shifted and Rotated Lunacek Bi-Rastrigin Function	Multimodal	700
F7	Shifted and Rotated Non-Continuous Rastrigin’s Function	Multimodal	800
F8	Shifted and Rotated Lévy Function	Multimodal	900
F9	Shifted and Rotated Schwefel’s Function	Multimodal	1000
F10	Hybrid Function 1 (N = 3)	Hybrid	1100
F11	Hybrid Function 2 (N = 3)	Hybrid	1200
F12	Hybrid Function 3 (N = 3)	Hybrid	1300
F13	Hybrid Function 4 (N = 4)	Hybrid	1400
F14	Hybrid Function 5 (N = 4)	Hybrid	1500
F15	Hybrid Function 6 (N = 4)	Hybrid	1600
F16	Hybrid Function 6 (N = 5)	Hybrid	1700
F17	Hybrid Function 6 (N = 5)	Hybrid	1800
F18	Hybrid Function 6 (N = 5)	Hybrid	1900
F19	Hybrid Function 6 (N = 6)	Hybrid	2000
F20	Composition Function 1 (N = 3)	Composition	2100
F21	Composition Function 2 (N = 3)	Composition	2200
F22	Composition Function 3 (N = 4)	Composition	2300
F23	Composition Function 4 (N = 4)	Composition	2400
F24	Composition Function 5 (N = 5)	Composition	2500
F25	Composition Function 6 (N = 5)	Composition	2600
F26	Composition Function 7 (N = 6)	Composition	2700
F27	Composition Function 8 (N = 6)	Composition	2800
F28	Composition Function 9 (N = 3)	Composition	2900
F29	Composition Function 10 (N = 3)	Composition	3000

**Table 2:** Hyperparameters for correlation algorithms.

Name	Parameters
ECPO	$p_{l}\;$= 0.3; $p_{e}\;$= 0.7
PO	$p_{l}\;$= 0.3; $p_{e}\;$= 0.7
SMA	/
WOA	$a_{1\;}$= [2, 0]; $a_{2}\;$= [−1, −2]; b = 1
MFO	b = 1; t = [−1, 1]; a = [−1, −2]
BA	/
SCA	/
PSO	$V_{max}$ = 6; $W_{max}$ = 0.9, $W_{min\;}$= 0.2; $C_{1\;}$= 2; $C_{2\;}$= 2
DE	a = [2, 0]

**Table 3:** Results of the ECPO and Other Algorithms on CEC2017.

	F1		F2		F3	
	Avg	Std	Avg	Std	Avg	Std
ECPO	2.5533 × 10^3^	3.3868 × 10^3^	6.0183 × 10^3^	1.7925 × 10^3^	**5.9406 × 10^2^**	2.1013 × 10^1^
PO	5.7678 × 10^7^	8.8266 × 10^7^	5.1121 × 10^3^	2.6765 × 10^3^	7.1852 × 10^2^	4.8687 × 10^1^
SMA	2.8682 × 10^9^	1.2223 × 10^9^	3.4641 × 10^4^	6.1441 × 10^3^	7.1538 × 10^2^	2.7779 × 10^1^
WOA	3.8858 × 10^6^	3.0510 × 10^6^	1.6617 × 10^5^	5.8485 × 10^4^	7.9399 × 10^2^	5.6422 × 10^1^
MFO	1.1190 × 10^10^	8.5527 × 10^9^	9.8579 × 10^4^	7.2925 × 10^4^	7.0977 × 10^2^	4.3436 × 10^1^
BA	5.3477 × 10^5^	3.4745 × 10^5^	3.0010 × 10^2^	9.9425 × 10^−2^	8.3536 × 10^2^	7.2939 × 10^1^
SCA	1.2294 × 10^10^	3.1232 × 10^9^	3.8025 × 10^4^	7.6942 × 10^3^	7.7882 × 10^2^	1.9855 × 10^1^
PSO	3.3674 × 10^3^	4.4518 × 10^3^	**3.0001 × 10^2^**	9.7402 × 10^−3^	6.9581 × 10^2^	3.2590 × 10^1^
DE	**1.6720 × 10^3^**	2.6872 × 10^3^	2.0113 × 10^4^	4.4064 × 10^3^	6.0597 × 10^2^	9.7270 × 10^0^
	F4		F5		F6	
	Avg	Std	Avg	Std	Avg	Std
ECPO	**5.9108 × 10^2^**	1.9276 × 10^1^	**6.0000 × 10^2^**	9.0390 × 10^−7^	8.4685 × 10^2^	2.9649 × 10^1^
PO	7.1669 × 10^2^	3.4384 × 10^1^	6.5577 × 10^2^	9.1330 × 10^0^	1.1843 × 10^3^	7.6114 × 10^1^
SMA	7.0915 × 10^2^	3.6763 × 10^1^	6.4378 × 10^2^	1.0139 × 10^1^	1.0658 × 10^3^	6.3361 × 10^1^
WOA	7.7040 × 10^2^	4.4565 × 10^1^	6.6792 × 10^2^	8.4931 × 10^0^	1.2419 × 10^3^	9.0990 × 10^1^
MFO	7.1352 × 10^2^	3.8297 × 10^1^	6.4130 × 10^2^	1.0526 × 10^1^	1.1420 × 10^3^	1.8908 × 10^2^
BA	8.3843 × 10^2^	6.8433 × 10^1^	6.7011 × 10^2^	9.2768 × 10^0^	1.5929 × 10^3^	2.0371 × 10^2^
SCA	7.7418 × 10^2^	1.5843 × 10^1^	6.5148 × 10^2^	6.4379 × 10^0^	1.1344 × 10^3^	4.4865 × 10^1^
PSO	6.9478 × 10^2^	3.5034 × 10^1^	6.4689 × 10^2^	8.2484 × 10^0^	1.0325 × 10^3^	8.5500 × 10^1^
DE	6.1027 × 10^2^	1.0950 × 10^1^	**6.0000 × 10^2^**	2.1111 × 10^−14^	**8.4277 × 10^2^**	1.0209 × 10^1^
	F7		F8		F9	
	Avg	Std	Avg	Std	Avg	Std
ECPO	**8.8811 × 10^2^**	2.1385 × 10^1^	1.0710 × 10^3^	2.8770 × 10^2^	**3.6511 × 10^3^**	3.7742 × 10^2^
PO	9.6243 × 10^2^	2.6318 × 10^1^	5.2394 × 10^3^	7.8276 × 10^2^	5.9115 × 10^3^	8.8067 × 10^2^
SMA	9.6665 × 10^2^	1.8766 × 10^1^	5.5914 × 10^3^	1.0315 × 10^3^	5.7496 × 10^3^	6.1702 × 10^2^
WOA	1.0067 × 10^3^	3.6604 × 10^1^	7.9806 × 10^3^	1.9843 × 10^3^	6.1472 × 10^3^	7.7219 × 10^2^
MFO	1.0103 × 10^3^	4.8478 × 10^1^	7.1955 × 10^3^	2.0629 × 10^3^	5.4253 × 10^3^	8.1241 × 10^2^
BA	1.0634 × 10^3^	5.3963 × 10^1^	1.3610 × 10^4^	4.9676 × 10^3^	5.6359 × 10^3^	6.5778 × 10^2^
SCA	1.0517 × 10^3^	1.8991 × 10^1^	5.2171 × 10^3^	1.3222 × 10^3^	8.1202 × 10^3^	2.1751 × 10^2^
PSO	9.5196 × 10^2^	3.4527 × 10^1^	4.3162 × 10^3^	9.8906 × 10^2^	5.2490 × 10^3^	4.9744 × 10^2^
DE	9.0921 × 10^2^	9.2515 × 10^0^	**9.0000 × 10^2^**	1.0125 × 10^−13^	5.8029 × 10^3^	2.6995 × 10^2^
	F10		F11		F12	
	Avg	Std	Avg	Std	Avg	Std
ECPO	1.1725 × 10^3^	3.3533 × 10^1^	3.6206 × 10^5^	2.1631 × 10^5^	1.7551 × 10^4^	1.9244 × 10^4^
PO	1.3148 × 10^3^	7.4800 × 10^1^	2.3973 × 10^7^	1.8828 × 10^7^	1.1801 × 10^5^	8.5025 × 10^4^
SMA	1.5223 × 10^3^	1.0297 × 10^2^	1.0611 × 10^8^	5.8804 × 10^7^	2.7739 × 10^6^	4.2840 × 10^6^
WOA	1.5213 × 10^3^	1.3839 × 10^2^	3.2133 × 10^7^	2.5845 × 10^7^	1.4900 × 10^5^	8.0362 × 10^4^
MFO	6.4286 × 10^3^	6.0657 × 10^3^	3.9567 × 10^8^	5.6595 × 10^8^	2.6205 × 10^8^	7.2166 × 10^8^
BA	1.3098 × 10^3^	5.8084 × 10^1^	1.6301 × 10^6^	1.1887 × 10^6^	2.9733 × 10^5^	1.3313 × 10^5^
SCA	2.1802 × 10^3^	6.1972 × 10^2^	1.1914 × 10^9^	2.9546 × 10^8^	4.5240 × 10^8^	3.4561 × 10^8^
PSO	1.2127 × 10^3^	3.7415 × 10^1^	**4.6061 × 10^4^**	2.6228 × 10^4^	**1.5307 × 10^4^**	1.3263 × 10^4^
DE	**1.1605 × 10^3^**	2.3085 × 10^1^	1.6235 × 10^6^	1.0534 × 10^6^	3.4063 × 10^4^	1.8594 × 10^4^
	F13		F14		F15	
	Avg	Std	Avg	Std	Avg	Std
ECPO	6.5336 × 10^4^	7.4352 × 10^4^	8.7271 × 10^3^	9.4149 × 10^3^	2.2824 × 10^3^	2.4337 × 10^2^
PO	3.9427 × 10^4^	2.5394 × 10^4^	6.3004 × 10^4^	7.0528 × 10^4^	3.0432 × 10^3^	4.0381 × 10^2^
SMA	1.7908 × 10^5^	8.5321 × 10^4^	1.7915 × 10^4^	9.2843 × 10^3^	2.8746 × 10^3^	3.6782 × 10^2^
WOA	7.3860 × 10^5^	7.4934 × 10^5^	7.8654 × 10^4^	5.8614 × 10^4^	3.6492 × 10^3^	5.8224 × 10^2^
MFO	1.5150 × 10^5^	3.2789 × 10^5^	3.0150 × 10^7^	1.6484 × 10^8^	3.1766 × 10^3^	4.0829 × 10^2^
BA	7.4300 × 10^3^	5.0607 × 10^3^	1.0372 × 10^5^	5.6221 × 10^4^	3.3258 × 10^3^	4.0253 × 10^2^
SCA	1.3310 × 10^5^	8.2141 × 10^4^	1.2124 × 10^7^	1.0113 × 10^7^	3.6278 × 10^3^	2.1015 × 10^2^
PSO	**5.8906 × 10^3^**	2.7806 × 10^3^	**6.2839 × 10^3^**	5.5652 × 10^3^	2.8517 × 10^3^	2.5253 × 10^2^
DE	4.4570 × 10^4^	2.5716 × 10^4^	7.5459 × 10^3^	4.6732 × 10^3^	**2.0702 × 10^3^**	1.9291 × 10^2^
	F16		F17		F18	
	Avg	Std	Avg	Std	Avg	Std
ECPO	1.9136 × 10^3^	1.2822 × 10^2^	3.7244 × 10^5^	3.4390 × 10^5^	7.0782 × 10^3^	8.3821 × 10^3^
PO	2.3801 × 10^3^	1.9388 × 10^2^	5.8504 × 10^5^	4.4892 × 10^5^	5.8349 × 10^5^	4.2013 × 10^5^
SMA	2.2758 × 10^3^	1.6430 × 10^2^	5.3866 × 10^5^	7.5932 × 10^5^	2.8899 × 10^5^	4.1376 × 10^5^
WOA	2.5498 × 10^3^	2.6048 × 10^2^	1.8399 × 10^6^	2.1360 × 10^6^	2.5475 × 10^6^	2.1432 × 10^6^
MFO	2.5970 × 10^3^	2.3042 × 10^2^	5.5814 × 10^6^	1.5538 × 10^7^	8.3761 × 10^6^	3.2804 × 10^7^
BA	2.7586 × 10^3^	2.6623 × 10^2^	**1.5614 × 10^5^**	1.2508 × 10^5^	6.8822 × 10^5^	2.2271 × 10^5^
SCA	2.4064 × 10^3^	1.3907 × 10^2^	3.0596 × 10^6^	1.5234 × 10^6^	2.2375 × 10^7^	1.0658 × 10^7^
PSO	2.4592 × 10^3^	2.9620 × 10^2^	1.8900 × 10^5^	1.5659 × 10^5^	**6.6243 × 10^3^**	5.6651 × 10^3^
DE	**1.8402 × 10^3^**	5.3057 × 10^1^	2.9290 × 10^5^	1.5946 × 10^5^	7.9477 × 10^3^	4.8951 × 10^3^
	F19		F20		F21	
	Avg	Std	Avg	Std	Avg	Std
ECPO	2.2751 × 10^3^	1.2262 × 10^2^	**2.3819 × 10^3^**	1.5295 × 10^1^	**2.3992 × 10^3^**	5.4327 × 10^2^
PO	2.5437 × 10^3^	1.2347 × 10^2^	2.4876 × 10^3^	6.8086 × 10^1^	3.5938 × 10^3^	2.0824 × 10^3^
SMA	2.4542 × 10^3^	1.2723 × 10^2^	2.4753 × 10^3^	2.6051 × 10^1^	4.0557 × 10^3^	2.1240 × 10^3^
WOA	2.6637 × 10^3^	2.2014 × 10^2^	2.5756 × 10^3^	6.2939 × 10^1^	7.4764 × 10^3^	1.6306 × 10^3^
MFO	2.6983 × 10^3^	2.4920 × 10^2^	2.5084 × 10^3^	3.9069 × 10^1^	6.5151 × 10^3^	1.4708 × 10^3^
BA	2.9531 × 10^3^	2.2637 × 10^2^	2.5920 × 10^3^	6.6446 × 10^1^	7.1956 × 10^3^	1.3313 × 10^3^
SCA	2.5736 × 10^3^	1.0855 × 10^2^	2.5513 × 10^3^	1.7852 × 10^1^	7.8573 × 10^3^	2.5756 × 10^3^
PSO	2.6252 × 10^3^	2.2683 × 10^2^	2.4644 × 10^3^	4.4789 × 10^1^	4.9218 × 10^3^	2.1152 × 10^3^
DE	2.1201 × 10^3^	**6.5962 × 10^1^**	2.4092 × 10^3^	8.7803 × 10^0^	4.3067 × 10^3^	2.0968 × 10^3^
	F22		F23		F24	
	Avg	Std	Avg	Std	Avg	Std
ECPO	**2.7283 × 10^3^**	1.8692 × 10^1^	**2.9069 × 10^3^**	2.4259 × 10^1^	2.8953 × 10^3^	1.8401 × 10^1^
PO	2.9626 × 10^3^	6.5892 × 10^1^	3.0979 × 10^3^	6.7596 × 10^1^	2.9305 × 10^3^	2.4406 × 10^1^
SMA	2.8616 × 10^3^	2.8845 × 10^1^	3.0110 × 10^3^	2.6314 × 10^1^	3.0129 × 10^3^	4.4877 × 10^1^
WOA	3.0630 × 10^3^	1.0752 × 10^2^	3.1687 × 10^3^	7.7939 × 10^1^	2.9545 × 10^3^	3.7077 × 10^1^
MFO	2.8320 × 10^3^	3.2325 × 10^1^	2.9960 × 10^3^	3.1654 × 10^1^	3.4894 × 10^3^	6.4121 × 10^2^
BA	3.3552 × 10^3^	1.2481 × 10^2^	3.3999 × 10^3^	1.3564 × 10^2^	2.9048 × 10^3^	2.3391 × 10^1^
SCA	2.9882 × 10^3^	2.4771 × 10^1^	3.1570 × 10^3^	2.2646 × 10^1^	3.1938 × 10^3^	7.2395 × 10^1^
PSO	3.2553 × 10^3^	1.4516 × 10^2^	3.3690 × 10^3^	1.0292 × 10^2^	**2.8797 × 10^3^**	4.1309 × 10^0^
DE	2.7578 × 10^3^	9.6283 × 10^0^	2.9551 × 10^3^	1.5861 × 10^1^	2.8874 × 10^3^	3.6306 × 10^−1^
	F25		F26		F27	
	Avg	Std	Avg	Std	Avg	Std
ECPO	**4.1785 × 10^3^**	7.8488 × 10^2^	3.2169 × 10^3^	1.1041 × 10^1^	3.1625 × 10^3^	4.9575 × 10^1^
PO	6.4763 × 10^3^	1.6166 × 10^3^	3.3401 × 10^3^	6.0505 × 10^1^	3.3120 × 10^3^	4.1035 × 10^1^
SMA	5.1692 × 10^3^	7.5012 × 10^2^	3.2677 × 10^3^	4.4365 × 10^1^	3.4222 × 10^3^	5.4394 × 10^1^
WOA	7.7623 × 10^3^	1.0328 × 10^3^	3.3540 × 10^3^	6.1127 × 10^1^	3.3084 × 10^3^	5.2989 × 10^1^
MFO	5.9229 × 10^3^	3.8663 × 10^2^	3.2597 × 10^3^	2.9353 × 10^1^	4.3155 × 10^3^	9.3149 × 10^2^
BA	9.0215 × 10^3^	2.3002 × 10^3^	3.4622 × 10^3^	1.3974 × 10^2^	**3.1250 × 10^3^**	5.2099 × 10^1^
SCA	6.9036 × 10^3^	3.2348 × 10^2^	3.3982 × 10^3^	3.9296 × 10^1^	3.8181 × 10^3^	1.4307 × 10^2^
PSO	6.8379 × 10^3^	2.3430 × 10^3^	3.3153 × 10^3^	3.2316 × 10^2^	3.1413 × 10^3^	5.2797 × 10^1^
DE	4.6352 × 10^3^	7.6760 × 10^1^	**3.2051 × 10^3^**	3.4090 × 10^0^	3.1925 × 10^3^	5.2602 × 10^1^
	F28		F29			
	Avg	Std	Avg	Std		
ECPO	3.6216 × 10^3^	1.6586 × 10^2^	9.5921 × 10^3^	4.2939 × 10^3^		
PO	4.5766 × 10^3^	3.6111 × 10^2^	5.6228 × 10^6^	3.4315 × 10^6^		
SMA	4.0411 × 10^3^	1.9995 × 10^2^	4.7570 × 10^6^	2.3763 × 10^6^		
WOA	4.8224 × 10^3^	4.7130 × 10^2^	1.0089 × 10^7^	6.4263 × 10^6^		
MFO	4.1861 × 10^3^	3.5153 × 10^2^	1.2444 × 10^6^	2.8259 × 10^6^		
BA	5.0852 × 10^3^	4.7170 × 10^2^	1.2750 × 10^6^	7.5539 × 10^5^		
SCA	4.6824 × 10^3^	2.5762 × 10^2^	7.7851 × 10^7^	3.2081 × 10^7^		
PSO	3.9629 × 10^3^	3.1779 × 10^2^	**6.5335 × 10^3^**	3.3407 × 10^3^		
DE	**3.5197 × 10^3^**	6.4547 × 10^1^	1.2726 × 10^4^	4.0079 × 10^3^		
	Overall Rank					
	RANK	+/=/−	AVG	Computational Time(s)		
ECPO	1	~	2.0345	183.16		
PO	5	26/3/0	5.1379	118.33		
SMA	4	28/1/0	4.9655	176.48		
WOA	8	29/0/0	7.1724	98.34		
MFO	6	28/1/0	6.3103	114.83		
BA	7	25/0/4	6.5517	116.22		
SCA	9	29/0/0	7.3448	106.56		
PSO	3	17/6/6	3.2069	91.75		
DE	2	14/8/7	2.2759	143.73		

**Table 4:** The *p*-values of the ECPO versus other algorithms on CEC2017.

	PO	SMA	WOA	MFO
F1	1.73 × 10^−6^	1.73 × 10^−6^	1.73 × 10^−6^	1.73 × 10^−6^
F2	1.53 × 10^−1^	1.73 × 10^−6^	1.73 × 10^−6^	6.98 × 10^−6^
F3	1.73 × 10^−6^	1.73 × 10^−6^	1.73 × 10^−6^	1.73 × 10^−6^
F4	1.92 × 10^−6^	1.73 × 10^−6^	1.73 × 10^−6^	1.73 × 10^−6^
F5	1.73 × 10^−6^	1.73 × 10^−6^	1.73 × 10^−6^	1.73 × 10^−6^
F6	1.73 × 10^−6^	1.73 × 10^−6^	1.73 × 10^−6^	1.73 × 10^−6^
F7	2.35 × 10^−6^	1.73 × 10^−6^	1.73 × 10^−6^	1.92 × 10^−6^
F8	1.73 × 10^−6^	1.73 × 10^−6^	1.73 × 10^−6^	1.73 × 10^−6^
F9	1.73 × 10^−6^	1.73 × 10^−6^	1.73 × 10^−6^	2.13 × 10^−6^
F10	1.73 × 10^−6^	1.73 × 10^−6^	1.73 × 10^−6^	1.73 × 10^−6^
F11	1.73 × 10^−6^	1.73 × 10^−6^	1.73 × 10^−6^	1.92 × 10^−6^
F12	1.73 × 10^−6^	1.73 × 10^−6^	1.73 × 10^−6^	1.73 × 10^−6^
F13	1.71 × 10^−1^	2.22 × 10^−4^	3.52 × 10^−6^	5.04 × 10^−1^
F14	6.34 × 10^−6^	7.71 × 10^−4^	2.13 × 10^−6^	2.35 × 10^−6^
F15	3.52 × 10^−6^	1.73 × 10^−6^	1.73 × 10^−6^	2.88 × 10^−6^
F16	1.73 × 10^−6^	2.60 × 10^−6^	1.73 × 10^−6^	1.73 × 10^−6^
F17	8.22 × 10^−2^	5.44 × 10^−1^	9.71 × 10^−5^	6.84 × 10^−3^
F18	1.73 × 10^−6^	3.52 × 10^−6^	1.73 × 10^−6^	2.35 × 10^−6^
F19	7.69 × 10^−6^	3.72 × 10^−5^	3.52 × 10^−6^	6.98 × 10^−6^
F20	1.36 × 10^−5^	1.73 × 10^−6^	1.73 × 10^−6^	1.73 × 10^−6^
F21	1.64 × 10^−5^	1.73 × 10^−6^	1.73 × 10^−6^	1.73 × 10^−6^
F22	1.73 × 10^−6^	1.73 × 10^−6^	1.73 × 10^−6^	1.92 × 10^−6^
F23	1.73 × 10^−6^	1.73 × 10^−6^	1.73 × 10^−6^	1.73 × 10^−6^
F24	5.31 × 10^−5^	1.92 × 10^−6^	3.52 × 10^−6^	3.88 × 10^−6^
F25	1.49 × 10^−5^	3.06 × 10^−4^	1.92 × 10^−6^	1.73 × 10^−6^
F26	1.73 × 10^−6^	3.18 × 10^−6^	1.73 × 10^−6^	4.73 × 10^−6^
F27	1.92 × 10^−6^	1.73 × 10^−6^	1.73 × 10^−6^	1.73 × 10^−6^
F28	1.73 × 10^−6^	3.52 × 10^−6^	1.73 × 10^−6^	5.22 × 10^−6^
F29	1.73 × 10^−6^	1.73 × 10^−6^	1.73 × 10^−6^	1.73 × 10^−6^
	BA	SCA	PSO	DE
F1	1.73 × 10^−6^	1.73 × 10^−6^	5.04 × 10^−1^	1.99 × 10^−1^
F2	1.73 × 10^−6^	1.73 × 10^−6^	1.73 × 10^−6^	1.73 × 10^−6^
F3	1.73 × 10^−6^	1.73 × 10^−6^	1.73 × 10^−6^	1.25 × 10^−2^
F4	1.73 × 10^−6^	1.73 × 10^−6^	1.73 × 10^−6^	5.29 × 10^−4^
F5	1.73 × 10^−6^	1.73 × 10^−6^	1.73 × 10^−6^	7.81 × 10^−3^
F6	1.73 × 10^−6^	1.73 × 10^−6^	1.73 × 10^−6^	7.66 × 10^−1^
F7	1.73 × 10^−6^	1.73 × 10^−6^	3.52 × 10^−6^	2.22 × 10^−4^
F8	1.73 × 10^−6^	1.73 × 10^−6^	1.73 × 10^−6^	8.21 × 10^−6^
F9	1.73 × 10^−6^	1.73 × 10^−6^	1.73 × 10^−6^	1.73 × 10^−6^
F10	1.73 × 10^−6^	1.73 × 10^−6^	4.20 × 10^−4^	1.36 × 10^−1^
F11	2.35 × 10^−6^	1.73 × 10^−6^	1.92 × 10^−6^	1.92 × 10^−6^
F12	1.73 × 10^−6^	1.73 × 10^−6^	7.81 × 10^−1^	6.64 × 10^−4^
F13	9.32 × 10^−6^	1.59 × 10^−3^	3.18 × 10^−6^	5.72 × 10^−1^
F14	1.73 × 10^−6^	1.73 × 10^−6^	2.99 × 10^−1^	7.66 × 10^−1^
F15	1.73 × 10^−6^	1.73 × 10^−6^	3.18 × 10^−6^	4.53 × 10^−4^
F16	1.73 × 10^−6^	1.73 × 10^−6^	1.73 × 10^−6^	2.70 × 10^−2^
F17	3.38 × 10^−3^	1.73 × 10^−6^	1.32 × 10^−2^	7.19 × 10^−1^
F18	1.73 × 10^−6^	1.73 × 10^−6^	6.00 × 10^−1^	5.19 × 10^−2^
F19	1.73 × 10^−6^	1.73 × 10^−6^	2.60 × 10^−6^	1.49 × 10^−5^
F20	1.73 × 10^−6^	1.73 × 10^−6^	1.92 × 10^−6^	3.18 × 10^−6^
F21	1.73 × 10^−6^	1.73 × 10^−6^	5.22 × 10^−5^	1.36 × 10^−5^
F22	1.73 × 10^−6^	1.73 × 10^−6^	1.73 × 10^−6^	8.47 × 10^−6^
F23	1.73 × 10^−6^	1.73 × 10^−6^	1.73 × 10^−6^	2.60 × 10^−6^
F24	4.07 × 10^−2^	1.73 × 10^−6^	1.49 × 10^−5^	1.25 × 10^−1^
F25	2.88 × 10^−6^	1.73 × 10^−6^	4.58 × 10^−5^	8.73 × 10^−3^
F26	1.73 × 10^−6^	1.73 × 10^−6^	5.71 × 10^−2^	5.79 × 10^−5^
F27	1.32 × 10^−2^	1.73 × 10^−6^	4.17 × 10^−1^	1.25 × 10^−2^
F28	1.73 × 10^−6^	1.73 × 10^−6^	5.31 × 10^−5^	4.11 × 10^−3^
F29	1.73 × 10^−6^	1.73 × 10^−6^	5.32 × 10^−3^	8.73 × 10^−3^

**Table 5:** Description of the experimental data sets.

Datasets	Samples	Features	Classes
Australian	690	14	2
cmc	1473	9	3
heartandlung	139	23	2
hepatitisfulldata	155	19	2
glass	214	9	6
heart	303	13	5
thyroid_2class	187	8	2
Leukemia	72	7130	2
Leukemia1	72	5327	3
M-of-n	1000	13	2

**Table 6:** Comparison results of the ECPO with other binary metaheuristic algorithms on KNN error rate.

Function	ECPO	BGWO	BGSA	BPSO	BBA	BSSA
Australian	**7.32 × 10^−2^**	8.00 × 10^−2^	8.37 × 10^−2^	7.97 × 10^−2^	2.36 × 10^−1^	7.82 × 10^−2^
	(2.46 × 10^−2^)	(2.38 × 10^−2^)	(2.16 × 10^−2^)	(1.97 × 10^−2^)	(8.46 × 10^−2^)	(3.72 × 10^−2^)
cmc	**4.54 × 10^−1^**	4.82 × 10^−1^	4.82 × 10^−1^	4.75 × 10^−1^	5.65 × 10^−1^	4.78 × 10^−1^
	(3.16 × 10^−2^)	(1.82 × 10^−2^)	(1.83 × 10^−2^)	(2.98 × 10^−2^)	(5.23 × 10^−2^)	(2.31 × 10^−2^)
heartandlung	**7.14 × 10^−3^**	1.44 × 10^−2^	1.49 × 10^−2^	1.43 × 10^−2^	1.59 × 10^−1^	1.67 × 10^−2^
	(2.29 × 10^−2^)	(3.13 × 10^−2^)	(3.02 × 10^−2^)	(2.36 × 10^−2^)	(1.21 × 10^−1^)	(3.02 × 10^−2^)
hepatitisfulldata	**5.36 × 10^−3^**	1.95 × 10^−2^	2.59 × 10^−2^	8.32 × 10^−3^	2.06 × 10^−1^	1.65 × 10^−2^
	(1.65 × 10^−3^)	(3.15 × 10^−2^)	(6.86 × 10^−3^)	(1.97 × 10^−3^)	(8.32 × 10^−2^)	(2.76 × 10^−3^)
glass	**9.86 × 10^−2^**	1.17 × 10^−1^	1.07 × 10^−1^	1.21 × 10^−1^	2.92 × 10^−1^	1.06 × 10^−1^
	(6.49 × 10^−2^)	(4.25 × 10^−2^)	(4.99 × 10^−2^)	(5.44 × 10^−2^)	(1.08 × 10^−1^)	(4.93 × 10^−2^)
heart	**4.72 × 10^−2^**	7.03 × 10^−2^	5.55 × 10^−2^	7.07 × 10^−2^	2.59 × 10^−1^	6.26 × 10^−2^
	(1.38 × 10^−2^)	(4.43 × 10^−2^)	(5.31 × 10^−2^)	(3.23 × 10^−2^)	(9.71 × 10^−2^)	(4.25 × 10^−2^)
thyroid_2class	**2.02 × 10^−1^**	2.01 × 10^−1^	2.14 × 10^−1^	2.08 × 10^−1^	3.21 × 10^−1^	2.17 × 10^−1^
	(6.72 × 10^−2^)	(6.49 × 10^−2^)	(6.85 × 10^−2^)	(7.31 × 10^−2^)	(8.81 × 10^−2^)	(7.59 × 10^−2^)
Leukemia	**0.00 × 10^0^**	**0.00 × 10^0^**	1.02 × 10^−1^	**0.00 × 10^0^**	1.67 × 10^−2^	**0.00 × 10^0^**
	(0.00 × 10^0^)	(0.00 × 10^0^)	(4.17 × 10^−2^)	(0.00 × 10^0^)	(5.21 × 10^−2^)	(0.00 × 10^0^)
Leukemia1	**0.00 × 10^0^**	**0.00 × 10^0^**	6.31 × 10^−2^	**0.00 × 10^0^**	5.19 × 10^−2^	**0.00 × 10^0^**
	(0.00 × 10^0^)	(0.00 × 10^0^)	(6.07 × 10^−2^)	(0.00 × 10^0^)	(6.05 × 10^−2^	(0.00 × 10^0^)
M-of-n	**0.00 × 10^0^**	**0.00 × 10^0^**	**0.00 × 10^0^**	**0.00 × 10^0^**	1.70 × 10^−1^	**0.00 × 10^0^**
	(0.00 × 10^0^)	(0.000 × 10^0^)	(0.00 × 10^0^)	(0.00 × 10^0^)	(9.05 × 10^−2^)	(0.00 × 10^0^)

**Table 7:** Comparison results of the ECPO with other binary metaheuristic algorithms on the number of features selected.

Function	ECPO	BGWO	BGSA	BPSO	BBA	BSSA
Australian	6.3	5.3	6.1	**5.1**	5.8	6.9
	(1.24)	(1.94)	(2.23)	(1.37)	(1.54)	(0.99)
cmc	5.3	**4.6**	6.3	5.1	5.7	5.3
	(0.823)	(1.08)	(0.99)	(0.87)	(1.31)	(0.94)
heartandlung	13.6	4.6	2.8	**2.** **6**	7.3	4.3
	(2.95)	(0.96)	(1.17)	(1.13)	(3.41)	(2.05)
hepatitisfulldata	10.2	4.3	**3.** **8**	6.1	7	5.4
	(2.52)	(1.15)	(1.70)	(2.02)	(2.82)	(2.59)
glass	5.4	3.8	4.6	3.9	**3.7**	4.2
	(0.96)	(0.63)	(1.42)	(0.73)	(1.56)	(0.78)
heart	8.1	5.9	6.5	5.6	**5.5**	6.4
	(1.10)	(1.19)	(1.26)	(1.64)	(1.50)	(1.71)
thyroid_2class	6.9	6.5	4.3	4	**3.7**	3.8
	(0.73)	(1.17)	(0.67)	(1.24)	(1.33)	(1.39)
Leukemia	2657.6	5356.5	**531.7**	2875	3240.3	2795.8
	(24.51)	(56.65)	(24.45)	(42.43)	(34.15)	(394.05)
Leukemia1	3425.4	4177.6	**377.1**	2092.7	2389.2	2215
	(50.57)	4(3.03)	(20.82)	(45.44)	(25.96)	(165.48)
M-of-n	8.2	**6**	**6**	**6**	7.9	6.1
	(0.84)	(0)	(0)	(0)	(1.72)	(0.31)

## Data Availability

The raw data supporting the conclusions of this article will be made available by the authors on request.
